# A Comparison between Decision Tree and Random Forest in Determining the Risk Factors Associated with Type 2 Diabetes

**Published:** 2018-04-24

**Authors:** Habibollah Esmaily, Maryam Tayefi, Hassan Doosti, Majid Ghayour-Mobarhan, Hossein Nezami, Alireza Amirabadizadeh

**Affiliations:** ^1^Social Determinants of Health Research Center, Mashhad University of Medical Sciences, Mashhad, Iran; ^2^Clinical Research Unit, Mashhad university of Medical Sciences, Mashhad, Iran; ^3^Department of Statistics, Macquarie University, Sydney, NSW, Australia; ^4^Biochemistry of Nutrition Research Center, School of Medicine, Mashhad University of Medical Sciences, Mashhad, Iran; ^5^Department of Modern Sciences and Technologies, School of Medicine, Mashhad University of Medical Sciences, Mashhad, Iran; ^6^Department of Basic Sciences, Faculty of Medicine, Gonabad University of Medical Sciences, Gonabad, Iran; ^7^Medical Toxicology and Drug Abuse Research Center (MTDRC), Birjand University of Medical Sciences, Birjand, Iran

**Keywords:** Diabetes mellitus, Decision tree, Random forest, data mining, Iran

## Abstract

**Background:** We aimed to identify the associated risk factors of type 2 diabetes mellitus (T2DM) using data mining approach, decision tree and random forest techniques using the Mashhad Stroke and Heart Atherosclerotic Disorders (MASHAD) Study program.

**Study design:** A cross-sectional study.

**Methods:** The MASHAD study started in 2010 and will continue until 2020. Two data mining tools, namely decision trees, and random forests, are used for predicting T2DM when some other characteristics are observed on 9528 subjects recruited from MASHAD database. This paper makes a comparison between these two models in terms of accuracy, sensitivity, specificity and the area under ROC curve.

**Results:** The prevalence rate of T2DM was 14% among these subjects. The decision tree model has 64.9% accuracy, 64.5% sensitivity, 66.8% specificity, and area under the ROC curve measuring 68.6%, while the random forest model has 71.1% accuracy, 71.3% sensitivity, 69.9% specificity, and area under the ROC curve measuring 77.3% respectively.

**Conclusions:** The random forest model, when used with demographic, clinical, and anthropometric and biochemical measurements, can provide a simple tool to identify associated risk factors for type 2 diabetes. Such identification can substantially use for managing the health policy to reduce the number of subjects with T2DM .

## Introduction

Type 2 diabetes mellitus (T2DM) is a major public health problem and its mortality is increasing worldwide^1,2^. WHO predicts the prevalence of T2DM in Iran to be 6.8% in 2025, and this translates to 5215000 citizens of Iran^3^.

The results of Tehran cohort show the prevalence of type T2DM in Iran is 11%^4^ and Mashhad cohort states this prevalence as 14% ^5^.

T2DM is one of the most serious challenges for developing countries in the 21^st^ century^6,7^. Diabetes has its roots in interactions between genetic, environmental and behavioral characteristics^8,9^. Cardiovascular diseases particularly are responsible for 80% of deaths due to T2DM^10^. Dominant possible risk factors in the development of T2DM are ethnicity, obesity, unhealthy diet, lack of physical activity, insulin resistance, and family history of diabetes^11^. Heart disease, stroke, blindness, kidney disease, and amputations are associated with diabetes ^12^. It is therefore essential to identify and diagnose individuals that run a high risk of T2DM ^6,13^.

In recent decades, different researchers in Iran have used data mining methods such as decision tree, neural network, support vector machine, random forest to predict the associated risk factors of T2DM^5,14^. One reason for not using classical statistical method is the number of predictors which the classical methods cannot select them conveniently. These two models, decision tree, and random forest are two of classification models and there are not so many studies in this regard.

Data mining is a new collection of statistical methods used to characteristics significantly associated with T2DM^15,16^. Data mining can discover new factors and also find relationships among factors that can reveal patterns and develop predictions based on new factors associated with T2DM^17,18^.

There are not many studies regarding associated risk factors of T2DM using data mining algorithms in Eastern Iran until yet. In this study, we developed the predicting model to identify associated risk factors of T2DM as a supplement in screening and public health in Eastern Iran.

## Methods

### Participants

The MASHAD study started in 2010 and will continue until 2020. The city of Mashhad is located in the north-eastern part of Iran. The total population of Mashhad was estimated using the national Iranian census of 2006 so the sample size was determined accordingly. Participants were enrolled from three regions of Mashhad. Each region was divided into nine sites centered at Mashhad Healthcare Center divisions. Overall, 9528 subjects were enrolled as a part of MASHAD study ^19^.

This protocol was approved by the Ethics Committee of MUMS, and an informed written consent was obtained from every participant.

Demographic characteristics such as age, gender, marital status, education, cigarette smoking habit, physical activity level (PAL), family history of diabetes (FHD) and depression score were collected from all the subjects. The Beck’s depression inventory-II (BDI-II) was used to evaluate the depression. Anthropometric information including weight, height, waist and hip circumference were obtained. Systolic and diastolic blood pressures were measured as described earlier ^19^. Biochemical parameters included: fasted serum triglycerides (TGs), total cholesterol (TC), HDL-cholesterol and LDL-cholesterol, fasting blood glucose (FBG) and hs-CRP were measured as previously described^19^. Diagnosed T2DM was identified based fasting blood glucose (FBG) ≥126 mg/dl ^20^.

### Input variables

The final data contains 9528 records and 18 variables, divided into 17 predictor variables and one outcome or target variable. The target variable has two possible states, namely occurrence of T2DM or no occurrence of T2DM. Demographic characteristics included age, gender, body mass index (BMI), marital status, level of education and biochemical markers, physical activity level (PAL), cigarette smoking habits, family history of diabetes (FHD) and depression score were considered as predictors (Table 1-2).

**Table 1:** Comparison of baseline characteristics between diabetes and non-diabetes groups

**Variables**	**Number**	**Percent**	**Number**	**Percent**	***P*** **value**
Sex					0.040
Male	518	38.1	3277	40.1	
Female	843	61.9	4890	59.9	
Educational level				0.001
High	109	8.0	936	11.5	
Moderate	374	27.5	2912	35.7	
Low	878	64.5	4319	52.9	
Occupational status				0.001
Employed	400	29.4	3114	38.1	
Retired	178	13.1	755	9.2	
Students	0	0.0	20	0.2	
Un employed	783	57.5	4278	52.4	
Marital status					0.001
Married	1239	91.0	7636	93.5	
Single	5	0.4	54	0.7	
Widow	96	7.1	366	4.5	
Divorced	21	1.5	111	1.4	
Smoking status					0.050
Yes	272	20.0	1775	21.7	
No	1089	80.0	6392	78.3	
Family history of diabetes				0.001
Yes	647	47.5	1994	24.4	
No	714	52.5	6173	75.6	
Depression					0.001
Yes	461	33.9	2226	27.3	
No	900	66.1	5941	27.7	

**Table 2:** Comparison of biochemical markers between diabetic and non-diabetic groups

**Variables**	**Diabetes**		**Non-diabetes**		***P*** **value**
	**Mean**	**SD**	**Mead**	**SD**	
Systolic blood pressure (mmHg)	128.8	18.4	121.1	18.2	0.001
Diastolic blood pressure (mmHg)	81.4	10.4	78.9	11.1	0.001
Total cholesterol (mg/dl)	205.5	46.3	189.7	37.8	0.001
Low-density lipoprotein (mg/dl)	122.5	39.1	115.7	34.6	0.001
High-density lipoprotein (mg/dl)	41.8	9.6	42.7	9.9	0.004
Triglycerides (mg/dl)	160.0	122.0	117.0	83.0	0.001
High-sensitivity -CRP	2.7	4.34	1.6	2.3	0.002

### Decision tree model

A decision tree is a non-parametric method named according to the nature of target variable. It is called a classification tree if the target variable is categorical and a regression tree if the target variable is continuous. The purpose of a decision tree is to develop a predictive model in terms of predictor variables. The tree is formed by successively dividing data according to one of the predictor variables. A decision tree consists of three types of nodes: root node, internal nodes, and leaf nodes^21-23^. Decision tree algorithms develop splitting criteria at internal nodes to from the tree. The split of a node attempts to minimize the impurity of the node. If a split is unable to achieve any improvement in terms of reducing impurity, the node is not split and is declared as a leaf node. If a split is able to reduce impurity, then the split providing the maximum reduction in impurity is selected and two branches are formed, forming two new nodes. The popular splitting criteria are information Gain, Gini index and gain ratio. CART is one of the decision tree algorithms that construct a binary tree using Gini index for selecting the splitting variable at every internal node. The Gini index at a node D is given by

$$\text{Gini}\left(\text{D}\right)=1-\overset{\text{m}}{\underset{\text{i}=1}{\sum }}{\text{P}}_{\text{i}}{}^{2}$$

where p_i_ is the probability that an observation in D belongs to the class Ci and is estimated by |Ci, D|/|D| ^24,25^. The sum is taken over them possible classes. The tree begins with all observations forming the root node and successive splits determine the order of importance of the predictor variables.

### Random Forest

Random forest is an ensemble learning method. It generates many classification trees by selecting subsets of the given dataset and selecting subsets of predictor variables randomly, finally aggregating the results of all models to obtain a random forest. Multiple classification trees are obtained from bootstrap samples in order to arrive at the final “majority” classification rules. The tree training parameters used in the present study are (i) ntree=500, the number of trees generated (ii) ntry=17, the number of predictor variables used in each tree, and (iii) node size=5, the minimum number of observations in a leaf node. Supervised machine learning algorithms divide the data into two parts, namely training data and test data.

One of the most important features of random forest and decision tree is the output of the variable importance. Variable importance measures the degree of association between a given variable and the classification result. Random forest and decision tree have four measures for the variable importance: raw importance score for class 0, raw importance score for class 1, decrease in accuracy and the Gini index^26^.

Statistical analyses were performed using R packages rpart (for decision trees), random Forest (for random forest) and caret. The complete sample contained 1361 individuals with T2DM and the remaining 8167 individuals without T2DM. The present study adopted a 10‐fold cross validation method to evaluate decision tree and random forest model. The 10‐fold cross validation method involves randomly separating the acquired data sets into 10 data sets that are equal in sample size. The decision tree and random forest models are constructed on the basis of a training data set. The rest of the nine data sets were used as testing data for verifying model effectiveness. Ten repeated empirical tests were conducted, where each subset was used as the test data. The bootstrap (500 replications) optimism-corrected area under the receiver operating characteristic curve (ROC) was estimated using R software.

The decision tree developed on the training data was used to obtain the splitting criteria for different nodes and was then applied to observation in the test data. The resulting tree is used to measure sensitivity, specificity, and accuracy of the model. If values of these measures are high for training data and lower for test data, it is considered as a case of overfitting. These measures must be obtained on training data as well as on test data in order to establish validity of the model. The models reported in this paper have been validated and results on test data are reported here.

Models are evaluated by constructing the confusion matrix for test data. In addition, accuracy, sensitivity, and specificity are also measured for each model. Accuracy, sensitivity, and specificity of a classification model are defined as follows ^27^.

- Accuracy=(TP+TN)/(TP+FP+TN+FN)
- Sensitivity=TP/(TP+FN)
- Specificity= TN/(FP+TN)

Here TP, TN, FP, and FN are truly positive, true negative, false positive and false negative respectively.

The receiver operating characteristic (ROC) curve is the plot that displays the full picture of trade-off between the sensitivity and (1- specificity) across a series of cutoff points. Area under the ROC curve is considered as an effective measure of inherent validity of a diagnostic test.

## Results

Anthropometric and biochemical features are summarized in Table 1 and 2, respectively. In general, 1361 (14.3%) people had T2DM. Of 1361 diabetic individuals, 843 (61.9%) were female, 1239 (91%) were married, and 783 (57.5%) were unemployed. Subjects with T2DM showed significantly higher systolic blood pressure, triglyceride, hs-CRP, diastolic blood pressure, serum total cholesterol, and LDL-cholesterol, whereas they showed significantly lower HDL-cholesterol than subjects without T2DM. The mean age of diabetic individuals was higher than non-diabetic individuals (52.01 ±7.2 vs 47.70 ±8.1, *P* <0.001). The mean BMI of diabetic patients was 28.78 ±4.4 and for non-diabetic persons was 27.76±4.7. The results of the independent *t* -test showed that the BMI in diabetics was significantly higher than non - diabetic people (*P* <0.001). The mean PAL of diabetic individuals was lower than non-diabetic individuals (1.59 ±0.86 vs 1.60 ±0.64, *P* =0.040).

**Figure 1:**
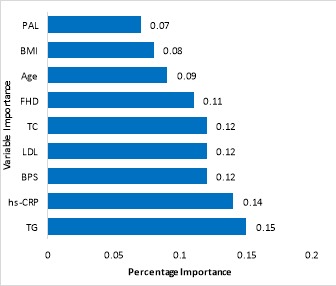
The importance of input variables in Random Forest model. X-axis shows the percentage of importance and the Y-axis represents the variables’ importance

Based on the results of the random forest model, TG, hs-CRP, SBP, LDL, TC, FHD, age, BMI, and PAL were the most important risk factors related to T2DM (Figure 1). In a subgroup with TG>204.5 and hs-CRP≤1.32 and occupation=employment, 79.2% was the probability of not occurrence of T2DM. In the subgroup with TG>204.5 and hs-CRP<1.32 and occupation=unemployment and hs-CRP>4.66, the probability of occurrence of T2DM is 90% (Table 3).

**Figure 2:**
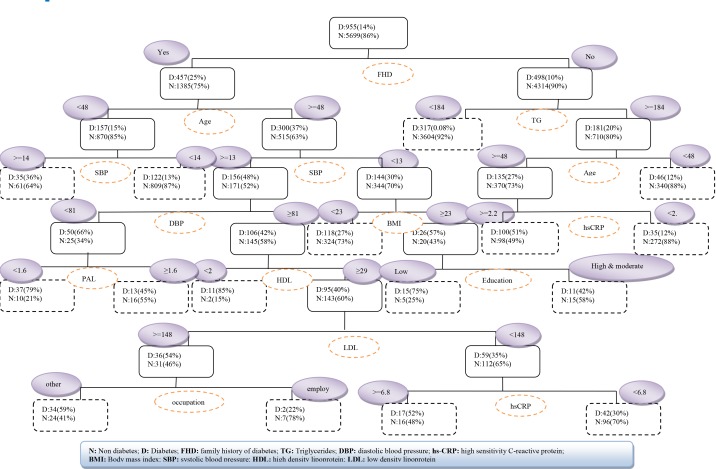
The decision tree CART with training dataset

Based on the results of the decision tree model, FHD, age, TG, SBP, hs-CRP, BMI, and DBP were the most important risk factors related to T2DM. Figure 2 shows the complete tree produced by CART. The decision tree showed that in a subgroup with FHD=no and TG<184, 92% is the probability of not occurrence of T2DM. In another subgroup, if FHD=yes, age<48 and SBP<140, the T2DM will not occur with probability of 87% (Table 3).

**Table 3:** The rules extracted through random forest and decision tree models

**Random forest model**
R1: IF TG>204.5 and hs-CRP≤1.32 and occupation=employment, THEN class: person without diabetes (187/236 or 79.2%) R2: IF TG>204.5 and hs-CRP<1.32 and occupation=retired and TC≤257, THEN class: person without diabetes (43/72 or 59.7%) R3: IF TG>204.5 and hs-CRP<1.32 and occupation=retired and TC>257 and LDL≤110.9, THEN class: person without diabetes (21/23 or 91.3%) R4: IF TG>204.5 and hs-CRP<1.32 and occupation=retired and TC>257 and LDL>110.9, THEN class: person with diabetes (5/9 or 55.5%) R5: IF TG>204.5 and hs-CRP<1.32 and occupation=unemployment and hs-CRP>4.66, THEN class: person with diabetes (9/10 or 90%) R6: IF TG>204.5 and hs-CRP<1.32 and occupation=unemployment and hs-CRP≤4.66 and BPD<57.9, THEN class: person without diabetes (138/199 or 69.3%) R7: IF TG>204.5 and hs-CRP<1.32 and occupation=unemployment and hs-CRP≤4.66 and BPD>57.9 and FHD=yes, THEN class: person with diabetes (14/16 or 87.5%) R8: IF TG>204.5 and hs-CRP<1.32 and occupation=unemployment and hs-CRP≤4.66 and BPD>57.9 and FHD=no, THEN class: person without diabetes (25/32 or 78.1%) R9: IF TG≤204.5 and hs-CRP>1.81 and age≤46.10, THEN class: person without diabetes (569/753 or 79.1%) R10: IF TG≤204.5 and hs-CRP>1.81 and age>46.10 and HDL>67.5 and TG>227 and BMI> 24.61, THEN class: person with diabetes (8/9 or 88.8%) R11: IF TG≤204.5 and hs-CRP>1.81 and age>46.10 and HDL>67.5 and TG>227 and BMI≤24.61, THEN class: person without diabetes (5/9 or 55.5%) R12: IF TG≤204.5 and hs-CRP>1.81 and age>46.10 and HDL>67.5 and TG≤227, THEN class: person without diabetes (11/12 or 91.6%) R13: IF TG≤204.5 and hs-CRP>1.81 and age>46.10 and HDL≤67.5 and PAL>2.18, THEN class: person without diabetes (129/136 or 94.8%) R14: IF TG≤204.5 and hs-CRP>1.81 and age>46.10 and HDL≤67.5 and PAL≤2.18 and BPS≤128.16, THEN class: person without diabetes (4/8 or 50%) R15: IF TG≤204.5 and hs-CRP>1.81 and age>46.10 and HDL≤67.5 and PAL≤2.18 and BPS>128.16, THEN class: person with diabetes (8/12 or 66.6%)
**Decision tree model**
R1: IF FHD=no and TG<184, THEN class: person without diabetes (3604/3921 or 92%) R2: IF FHD=no, TG≥184 and age<48, THEN class: person without diabetes (340/386 or 88%) R3: IF FHD=no, TG≥184, age≥48 and hs-CRP<2.2, THEN class: person without diabetes (272/307 or 88%) R4: IF FHD=no, TG≥184, age≥48 and hs-CRP≥2.2, THEN class: person with diabetes (100/198 or 51%) R5: IF FHD=yes, age<48 and SBP<140, THEN class: person without diabetes (809/894 or 90%) R6: IF FHD=yes, age<48 and SBP≥140, THEN class: person with diabetes (72/133 or 54%) R7: IF FHD=yes, age≥48, SBP≥130, DBP<81 and PAL≥1.6, THEN class: person without diabetes (16/29 or 55%) R8: IF FHD=yes, age≥48, SBP≥130, DBP<81 and PAL<1.6, THEN class: person with diabetes (37/47 or 79%) R9: IF FHD=yes, age≥48, SBP≥130, DBP≥81, HDL<29, THEN class: person with diabetes (11/13 or 85%) R10: IF FHD=yes, age≥48, SBP≥130, DBP≥81, HDL≥29, LDL<148 and hs-CRP<6.8, THEN class: person without diabetes (96/138 or 70%) R11: IF FHD=yes, age≥48, SBP≥130, DBP≥81, HDL≥29, LDL<148 and hs-CRP≥6.8, THEN class: person with diabetes (17/33 or 52%) R12: IF FHD=yes, age≥48, SBP≥130, DBP≥81, HDL≥29, LDL≥148 and occupation=employed, THEN class: person without diabetes (7/9 or 78%) R13: IF FHD=yes, age≥48, SBP≥130, DBP≥81, HDL≥29, LDL≥148 and occupation=other, THEN class: person with diabetes (34/58 or 59%) R14: IF FHD=yes, age≥48, SBP<130, BMI<23, THEN class: person without diabetes (324/442 or 73%) R15: IF FHD=yes, age≥48, SBP<130, BMI≥23 and education=low, THEN class: person with diabetes (15/20 or 75%) R16: IF FHD=yes, age≥48, SBP<130, BMI≥23 and education=high &moderate, THEN class: person without diabetes (15/26 or 58%)

**Figure 3:**
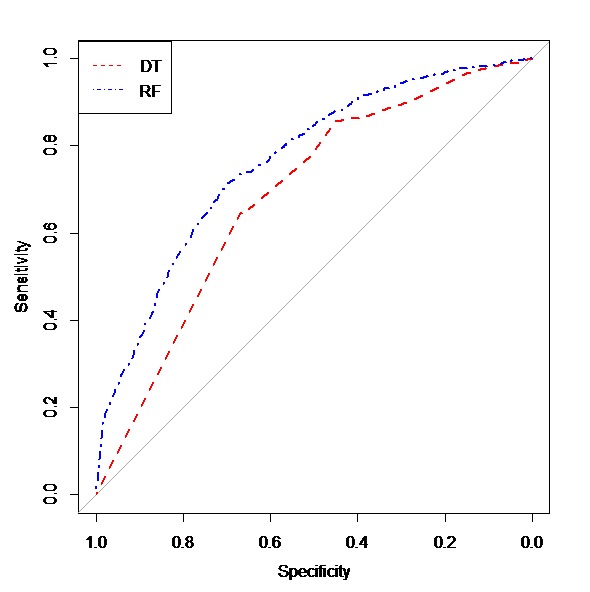
Roc curve of the DT and RF model in testing dataset

Sensitivity (95% CI) of decision tree and random forest model are, respectively, 64.5% (62.9, 86.7) and 71.3% (65.3, 74.4), and their specificity (95% CI) rate are 66.8% (58.3, 70.8) and 69.9% (65.4, 77.1) respectively, and their accuracy (95% CI) are 64.9% (63.6, 80.4) and 71.1% (66.8, 73.5). We used the area under curve ±standard error (95% CI) to compare these two models. The related value in the case of decision tree amounted to 68.6 ±1.39 (65.8-71.3) and 77.3±0.001 (73.8, 78.8) for the random forest model (Figure 3). The decision tree and random forest model (D=6.53, *P* <0.001).

## Discussion

We developed a prediction model based on cross-sectional study to predict risk factors of T2DM according to decision tree and random forest models.

The random forest model showed that TG, hs-CRP, SBP, LDL, TC, FHD, age, BMI, and PAL were strongly associated with T2DM. The decision tree model found FHD, age, TG, SBP, hs-CRP, BMI, and DBP were strongly associated with occurrence of T2DM. Putting the two results together, TG, FHD, hs-CRP, SBP, and BMI are common associated risk factors of T2DM in the two models. In a cohort study by using a decision tree, TG, family history of T2DM, BMI, SBP, education level and occupation were the associated risk factors of T2DM^4^.

Decision tree algorithm is a classification model based on different predictor variables and is widely being used in medicine^28-30^. RF creates multiple classification and regression (CART) trees, each trained on a bootstrap sample of the original training data and searches across a randomly selected subset of input variables to determine the split^31^. The variables such as family history of diabetes, age, triglycerides, LDL-cholesterol, body mass index, and physical activity level have already been identified as important associated risk factors of diabetes^32-34^. The present study has found hs-CRP as an important associated risk factor of T2DM, but it has not been reported so far ^28,33^.

The results of our study showed that family history of diabetes and triglycerides were the most important risk factors related to T2DM in the decision tree and random forest models. In other studies also, family history of diabetes and TG were the most important associated risk factors for T2DM^4,30^.

Decision trees are one of the easiest tools to decision systems and easy to understand. Decision trees can easily convert to if-then rules. Programs based on these rules can be made and used on personal computers for decision analyses, used easily with physicians and health care personnel to conclude the outcomes ^4,35-38^.

In this study, comparison of decision tree and random forest models showed that sensitivity and specificity values of random forest were higher than decision tree which was inconsistency with previous studies ^31,39^. On the other hand, sensitivity of C4.5 algorithm was higher than random forest, but specificity of random forest was higher than decision tree (C4.5)^39^. The reason for being difference between sensitivity of them is using different algorithm.

The ROC curve is a technique to visualize, organize, and choose classification based on the performance of the classification. The area under the curve (AUC) is an index of which model performs better and has a high level of accuracy. This index, which compares the performance of true positive and false positive of two different decision extremes, is often used to evaluate the predictive accuracy of classification models^40^.

In the current study, the AUC of random forest of testing dataset was significantly higher than decision tree which was consistent with previous studies ^31,39^. Random forest model is an accurate model for investigation of novel predictor markers, which is in line with previous^14,31^.

The strength of the study lies in its large sample size that makes it applicable to general population. One potential limitation of this study is that it is based on a cross-sectional data and cannot obtain results obtained from longitudinal or cohort data.

## Conclusions

Random forest models can provide good prediction models due to their efficacy and sensitivity and specificity. According to random forest model, TG and hs-CRP are the most important associated risk factors for T2DM. This study has also identified some new risk factors associated with T2DM indicating the need for further evaluation of clinical applicability of this model.

## Acknowledgements

This study was financially supported by the Biochemistry of Nutrition Research Center of Mashhad University of Medical Sciences, Mashhad, Iran.

## Conflict of interest statement

The authors declare that there is no conflict of interest.

## Funding

This study was supported by Mashhad university of medical sciences.

## Highlights

- Based on the RF model TG, hs-CRP, SBP, and FHD are the most important associated risk factors for T2DM.
- Based on the DT model FHD, TG, age, and hs-CRP are the most important associated risk factors for T2DM.
- RF model demonstrated a better discriminatory power compared with DT model.
